# The characterization of the transit through the anaerobic threshold based on relationships between RR and QRS cardiac intervals

**DOI:** 10.1371/journal.pone.0216938

**Published:** 2019-05-15

**Authors:** Loreta Saunoriene, Vaiva Siauciunaite, Alfonsas Vainoras, Virginija Bertasiute, Zenonas Navickas, Minvydas Ragulskis

**Affiliations:** 1 Center for Nonlinear Systems, Kaunas University of Technology, Studentu 50-146, Kaunas LT-51368, Lithuania; 2 Institute of Cardiology, Lithuanian University of Health Sciences, Sukileliu 17, Kaunas LT-50161, Lithuania; Sao Paulo State University (UNESP), BRAZIL

## Abstract

This paper aims to develop a novel computational technique for the detection of the transit through the anaerobic threshold. This technique uses only cardiac intervals derived from the electrocardiogram and is based on algebraic relationships between RR and QRS intervals. Electrocardiograms are measured during the load and the recovery processes. Algebraic relationships between cardiac intervals are used not only to identify the anaerobic threshold but also to characterise individual features of the person during the transit through the threshold. The ratio between carbon dioxide and oxygen in the exhaled air is used to validate the results. The algebraic relationship between cardiac intervals serves as a stand-alone indicator for both the determination of the anaerobic threshold and the characterization of the performance of the person during the load and the recovery processes.

## Introduction

Anaerobic threshold (AnT) is a frequently used standard term in sports medicine. The AnT is a reliable and powerful predictor of performance under the load. The AnT is sometimes defined equivalently to the lactate threshold (LT). The LT describes the exercise intensity beyond which blood lactate concentration is no longer linearly related to exercise intensity—but increases with both exercise intensity and duration [1]. The blood lactate concentration at the AnT is called the “maximum steady-state lactate concentration” (MLSS) [2]. The AnT is the point at which increased carbon dioxide production and minute ventilation result from increased levels of lactic acid during exercise. And although the LT and the AnT occur together under most conditions, strictly speaking even these two terms are not the same [3].

Several different validated methods are used to determine the AnT. Blood lactate accumulation still remains a good marker for the onset of fatigue—therefore blood lactate accumulation and maximal lactate steady state can be effectively used for the determination of the LT (and approximation of the AnT). The most accurate way to determine the LT is via a graded exercise test in a laboratory setting [4]. The resistance of the cycle ergometer is increased at regular intervals (typically every 1 min) and blood samples are taken at each increment. The lactate performance curve is then produced by plotting the blood lactate against each workload interval. The AnT can be determined from the lactate curve. A sudden or sharp rise in the curve above the base level is said to indicate the AnT [4]. However, it is often difficult to identify this sudden rise of the blood lactate [4].

In 1982 Conconi [5] stated that the AnT correlates to a deflection point in the heart rate during the exercise. The Conconi test is based on the assumption that the heart rate and the exercise intensity are linearly correlated (the heart rate increases at increasing intensities of the exercise). However, Conconi did demonstrate that the heart rate reached a plateau at near maximal exercise intensities in all their tested subjects [6]. The attractiveness of the Conconi test is firstly based on its simplicity. However, the accuracy of the Conconi test is seriously questioned by other researchers [7–9]. Studies have found that the deflection point or plateau in heart rate only occurs in a certain number of individuals and that when it does, it significantly overestimates directly measured the LT. Conconi and co-workers do acknowledge this controversy themselves [10]. Many other subsequent studies both support and contradict the applicability of the Conconi test [11, 12].

Often VO2 max, maximum heart rate and other physiological kinetics are measured during the same exercise [1]. However, the heart rate is never completely reliable and varies greatly between and within individuals during the exercise [1, 3, 4]. The combination of the heart rate with lactate measurements is considered more reliable than using just the Conconi test [5].

A very simply method for estimating the AnT is to assume that the AnT occurs when the heart rate reaches 85-90% of its maximum during the exercise (for 20-age healthy subjects). However, as mentioned earlier, the heart rate varies greatly between individuals and even within the same individual so this cannot be considered as a reliable test.

Some researchers have questioned the validity of determining the LT and the AnT even in laboratory settings [2, 13]. Yet more researchers question whether a definite point or threshold exists at all [13–16]. Instead they suggest blood lactate accumulation is continuous in nature and no specific threshold point can be determined at all.

It is a common agreement that any physiological test is only as reliable as the tester’s ability to follow a set protocol. Even when a suitable assessment has been chosen, numerous variables must be kept constant for any test to remain accurate and reliable.

That said, we do introduce two main objectives of this manuscript. Firstly, we will introduce a computational technique for the identification of the AnT from the relationships between RR and QRS duration cardiac intervals. Secondly, we will demonstrate how the presented technique is capable not only to identify the AnT, but also to characterize individual features of the transit through the AnT.

## Methods

The research met all applicable standards for the ethics of experimentation. Permit to perform biomedical investigation was granted by Kaunas Regional Ethics Committee for Biomedical Investigations, No. BE-2-51, 23.12.2015. ECG cycle ergometry exercise was used to record cardiac RR and QRS duration intervals. Participants provided written informed consent prior to the experiment.

### The description of the experimental setup

The measurement of cardiac intervals was performed by the ECG analysis system “Kaunas-Load” [17–20] developed at the Institute of Cardiology, Lithuanian University of Health Sciences. QRS duration interval was determined as heart ventricles depolarization time measured on ECG II lead.

Several clinical trials were used to assess and validate the accuracy of the detection of cardiac intervals during the development of “Kaunas-Load” system [18]. The cycle ergometry exercise is used for generating increasing physical loads. “Kaunas-Load” system synchronously registers 12 different standard parameters of the ECG. Initially, the measurement is started at zero load and continued for one minute. Then, the load is increased every consecutive minute by 25W. The person is asked to maintain a constant 60 revolutions per minute spinning rate during the whole cycle ergometry exercise. The exercise is continued until the first clinical indications for load limitation are observed according to AHA (American Heart Association). The measurement is then continued for another 10 minutes throughout the recovery process.

In parallel, systolic and diastolic pressures are measured and recorded at the middle of every minute throughout the experiment. Also, the ratio between carbon dioxide and oxygen in the exhaled air is measured during the cycle ergometry exercise and the recovery process.

The cohort comprised 9 healthy physically active men, not professional sportsmen. The mean and standard deviation of age is 44.78±12.08 years; height 1.809±0.085 m; weight 83.78±16.6 kg; body mass index 25.47±3.62 kg/m^2^.

### The description of the algorithm

Two ECG parameters (RR and QRS duration intervals) are continuously measured throughout the experiment and denoted as vectors *x* = (*x*_1_, *x*_2_, …, *x*_*n*_) and *y* = (*y*_1_, *y*_2_, …, *y*_*n*_) accordingly. The relationship between time series *x* and *y* is assessed using the computational technique presented in [21]. The algorithm comprises three basic parts.

1. First of all, elements of time series *x* and *y* are embedded into a sequence of perfect matrices of Lagrange differences $L_{\delta ,k}=[\begin{array}{cc} x_{k} & x_{k+\delta }-y_{k+\delta }\\ x_{k-\delta }-y_{k-\delta } & y_{k} \end{array}]$; *k* = (1 + *δ*), (2 + *δ*), …, (*n* − *δ*); *δ* ∈ *N* (the concept of a perfect matrix of Lagrange differences in introduced in [21]). From the topological point of view, two scalar trajectories are embedded into one three dimensional trajectory matrix. Note that a fixed matrix architecture (*L*_*δ*,*k*_) is used throughout all computational experiments.
2. The sequence of matrices *L*_*δ*,*k*_ is transformed into a scalar sequence of maximum modulus values of eigenvalues of matrices *L*_*δ*,*k*_; this scalar sequence is denoted as *s*_*δ*,*k*_ = max|λ (*L*_*δ*,*k*_)|. Note that a fixed matrix parameter (the maximum modulus value of two eigenvalues of *L*_*δ*,*k*_) is used throughout all computational experiments.
3. Finally, internal and external smoothing is applied for *s*_*δ*,*k*_. Let us denote the radius of the internal smoothing as *R*_*i*_; the radius of the external smoothing—as *R*_*e*_ (*R*_*i*_, *R*_*e*_ ∈ *N*). Then, the smoothed sequence reads [21]:
  $$\begin{array}{c} p_{k}\;(R_{i},R_{e})=\frac{1}{(2R_{e}+1)\;R_{i}}\sum_{j=k-R_{e}+1}^{k+R_{e}-1}\sum_{\delta =1}^{R_{i}}s_{\delta ,j}. \end{array}$$ (1)

Note that the computation of *p*_*k*_ (*R*_*i*_, *R*_*e*_) requires multiple embeddings of *x* and *y* into different trajectory matrices. The parameter sequence *p*_*k*_ (*R*_*i*_, *R*_*e*_) is used to assess the relationship between scalar sequences of cardiac intervals RR and QRS which are denoted as vectors *x* and *y* accordingly.

A well-posed optimization problem is formulated in [21] in respect of parameters *R*_*i*_, *R*_*e*_ (assessing the whole cohort of persons). We do not repeat the optimization procedures and keep *R*_*i*_ = 3 and *R*_*e*_ = 4 fixed in all further computations. The algorithm used for the investigation of relationships between RR and QRS duration intervals is kept the same as in [21]—except that now *y* represents the sequence of QRS duration intervals.

### Blood pressure and CO_2_/O_2_ ratio

Apart from the measurement of ECG, systolic and diastolic blood pressures and the proportion between the concentrations of carbon dioxide and oxygen in the exhaled air are measured throughout the cycle ergometry exercise and the recovery process.

Systolic and diastolic pressures are measured once per minute during the whole experiment. The ratio between carbon dioxide and oxygen in the exhaled air is measured continuously (the person wears a mask with the online gas analyzer)—but the results are averaged for every consecutive minute throughout the experiment. The averaging (one reading per minute) is performed in order to eliminate the effect of the variable exhale rate during the exercise. Also, as mentioned in the Introduction, the ratio between carbon dioxide and oxygen in the exhaled air is really an approximate measure of the AnT and such resolution in time is sufficient for our purposes.

## Results

### Experiment #1

**Fig 1:**
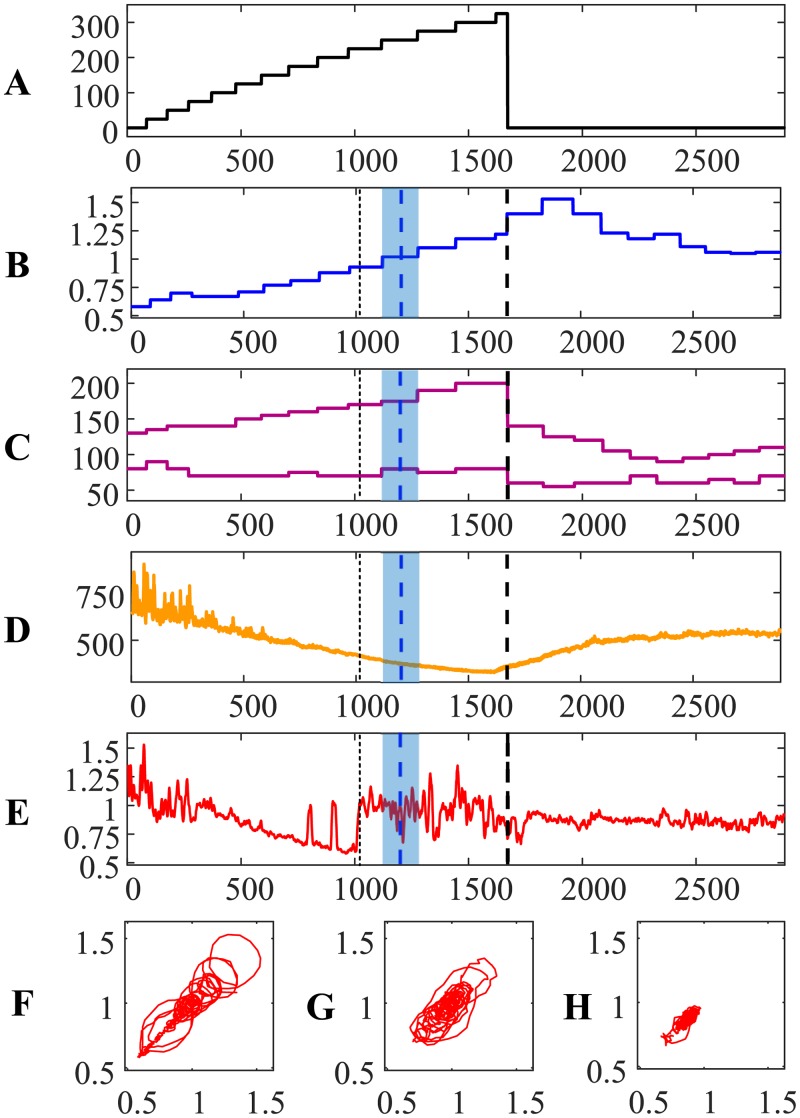
The transit through the AnT—Person #1. The *x*-axis in parts (A)–(E) represents the number of cardiac cycles from the beginning of the cycle ergometry exercise. (A) The power of the load in Watts. (B) The ratio between carbon dioxide and oxygen concentrations in the exhaled air. (C) Systolic and diastolic pressures in mmHg. (D): The variation of the RR interval in ms. E: The relationship between RR and QRS duration intervals. (F), (G) and (H): Attractors before the AnT, after the AnT but before the termination of the load, and during the recovery process. Thin vertical dotted line denotes the AnT based on the ECG. Thick vertical blue dashed line denotes the AnT based on the exhaled air. Thick vertical black dashed line denotes the termination of the load.

The evolution of the relationship between RR and QRS intervals during the load and the recovery processes for the first person is visualized in Fig 1. The *x*-axis in parts A–E represents the number of cardiac cycles from the beginning of the cycle ergometry exercise. The power of the load in Watts is shown in Fig 1A. The person is at rest at the beginning of the experiment for 1 minute (Fig 1A). Then, the load is increased by 25 W for each consecutive minute. The cycle ergometry exercise is continued for 13 minutes and is terminated in the middle of the 14th minute at the maximal load of 325 W (Fig 1). Note that the length of intervals in Fig 1A are not equal due to the increasing heart rate (different numbers of cardiac cycles fit into each minute). The recovery processes are recorded for 10 minutes after the termination of the cycle ergometry exercise.

The ratio between carbon dioxide and oxygen concentrations in the exhaled air (during the cycle ergometry exercise and the recovery process) is depicted in Fig 1B (the blue solid line). The concentrations are measured once per minute—therefore the blue solid line is a stepped line. As mentioned previously, the AnT can be marked at the moment when the ratio between carbon dioxide and oxygen in the exhaled air becomes equal to 0.95. The AnT is marked by a vertical dashed blue line at the center of the appropriate interval; the whole interval is depicted using a light blue background (Fig 1B). Also, the moment of the load termination is marked using a thick vertical dashed black line (throughout parts A–E in Fig 1).

Systolic and diastolic blood pressures (measured once per minute) are depicted in Fig 1C. Blood pressure data is not used in the algorithm for the detection of the AnT—it only shows the individual reactions of the cardiovascular system during the bicycle ergometry exercise.

The variation of the RR interval during the load and the recovery processes is illustrated in Fig 1D. This variation represents a continuous increase of the heart rate during the load. Also, Fig 1D is a good illustration of the effect of the collapse of complexity before reaching the maximal load [21]. However, there are no clear indicators in the RR interval time series which would denote the AnT.

The algebraic relationship between RR and QRS duration intervals is represented by the thin solid red line in Fig 1E. It is important to note that the effect of the collapse of complexity represented by this relationship happens not at the moment of termination of the cycle ergometry exercise (as demonstrated in [21])—but right at the AnT (Fig 1E). Moreover, it is interesting to observe that the cardiovascular system starts to seek for a new state before the AnT (observe two intermittent peaks in Fig 1E)—but the transition to the new state appears to happen too early—and the system returns to the regime of the gradual collapse of complexity.

The relationship between RR and QRS duration intervals exhibits a transition to a completely different state just before the AnT (Fig 1E). This transition can be explained by the necessity to find a new pattern of the self-organization of the cardiac system—what helps to open new functional possibilities to execute under the heavy physical load. The cardiac system continues to operate at this new state during the rest part of the load.

We postulate that the moment of this transition to the new state of self-organization is the moment of the AnT. It is important to note that the identification of this moment requires only ECG parameters—no other measurements are required for that purpose. This moment is depicted by a thin vertical dotted line (Fig 1E). Clearly, the time moments of the AnT identified from the ECG and from the exhaled air are different and do not coincide (Fig 1E). This is not surprising. The AnT reconstructed from the ECG represents the self-organization of the cardiovascular system—while the AnT reconstructed from the exhaled air represents the changes in the breathing system. Nevertheless, it is interesting to measure the difference between the time moments identified by two different methods. The AnT reconstructed from the ECG occurs 69.411 s earlier that the AnT reconstructed from the exhaled air (note that heartbeats are converted to seconds). It is logical to express this time difference in minutes (-1.1569 min) as the concentrations of gasses in the exhaled air are measured once per minute only. Note the negative sign which denotes that the AnT from the ECG occurred earlier than the AnT from the exhaled air.

The dynamics of the cardiac system in the process of the approach to the collapse of complexity before the anaerobic threshold can be visualized in the phase-plot diagram in Fig 1F. We use a standard two-dimensional embedding procedure with a near-optimal time delay to reconstruct the attractor [21]. Note that the observation window used to plot the attractor in Fig 1F covers the time segment from the beginning of the experiment to the AnT (reconstructed from the ECG).

Analogously, the attractor in Fig 1G is reconstructed from the time segment between the AnT and the end of the load. Finally, the attractor in Fig 1H represents the recovery process.

The geometric shape of the attractor in Fig 1F illustrates a gradual loss of the complexity of the cardiac system and the beginning of the process of wandering towards the new state. The attractor in Fig 1G represents the fluctuating dynamics in the new post-anaerobic state. The attractor in Fig 1H represents the calm recovery process.

We postulate that such a transition through the AnT is typical for a healthy person. In fact, the variation of systolic and diastolic pressures during and after the exercise (Fig 1C) indicates that the person could execute the cycle ergometry exercise without any problems. Diastolic blood pressure dropped at the beginning of the physical load; it did not exceed the initial reading until the end of the ergometry exercise. Systolic blood pressure did increase continuously during the ergometry exercise but did not exceed 200 mmHg at the termination point. Both systolic and diastolic pressures dropped right after the termination of the exercise and normalized during the recovery process.

### Experiments #2—#9

A number of experiments with other persons are performed in order to demonstrate the applicability of the introduced algorithm for the identification of the AnT based on the ECG cardiac parameters (RR and QRS duration). Also, that allows to observe and to compare the transient behavior of the cardiovascular system and its self-organization during the transit through the AnT.

**Fig 2:**
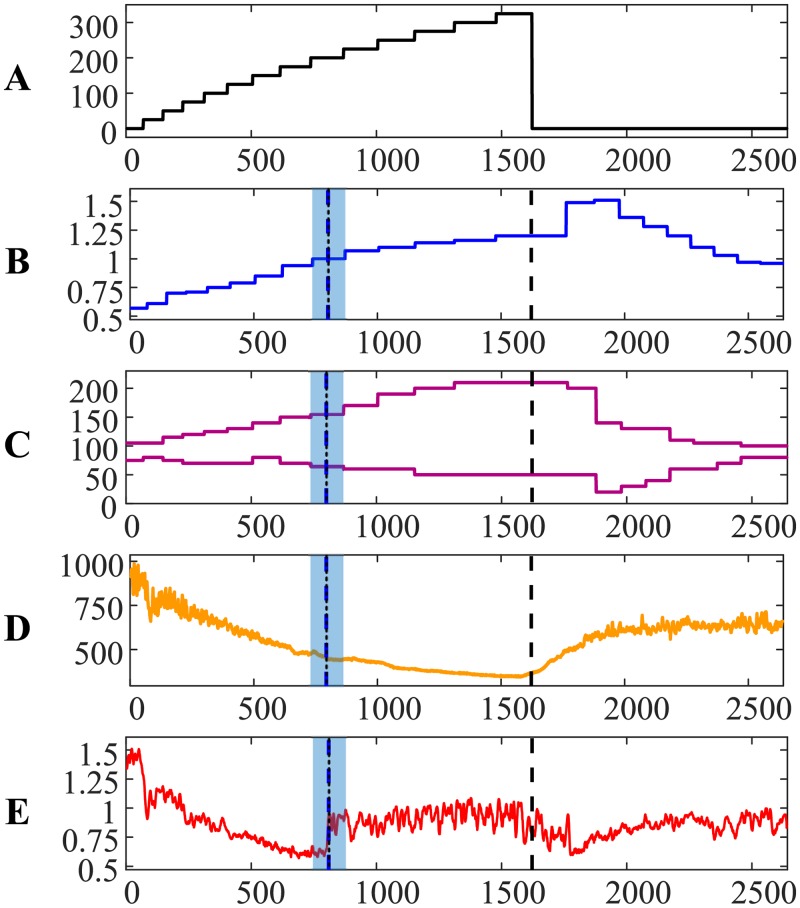
The transit through the AnT—Person #2.

The transit through the AnT for person #2 is depicted in Fig 2. Note that the AnT for person #2 occurred much earlier if compared to person #1. The relationship between RR and QRS duration intervals exhibit almost monotonous decline; no intermittent peaks are present before the AnT (Fig 2E). However, systolic and diastolic pressures did not drop immediately after the termination for the load (Fig 2). It is also interesting to observe that the reconstructed time moments of the AnT based on ECG and the exhaled air almost coincide—the difference is 0.0695 min. The comparison between Figs 2 and 1 reconfirms that each person is individual, and that the self-organization of each organism can be very distinctive.

**Fig 3:**
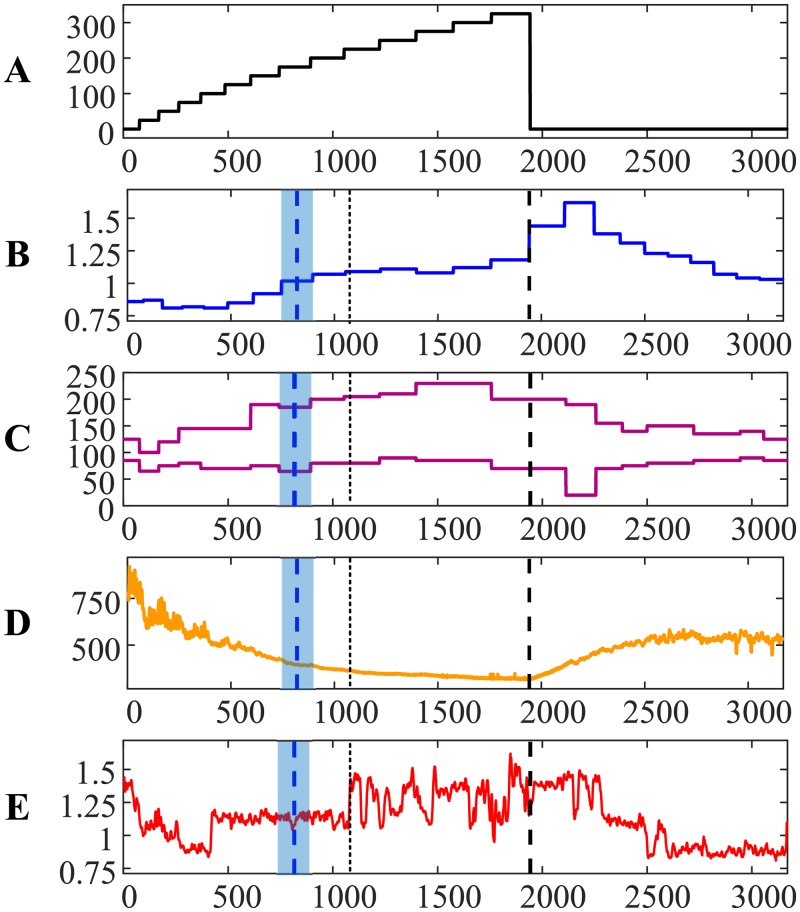
The transit through the AnT—Person #3.

The results for person #3 demonstrate yet another feature—the AnT based on ECG occurs much later than the AnT based on the exhaled air (Fig 3)—the difference is 1.6844 min. The relationship between RR and QRS duration intervals exhibits a small jump just before the 500-th contraction (Fig 3E)—but the cardiac system remains stable until the AnT. After the AnT, the relationship shows wild oscillations (similar to persons #1 and #2). However, the loss of stability of the relationship between RR and QRS duration intervals right after the 1000-th contraction (Fig 3E) is in a stark contrast to the dynamic behavior for persons #1 and #2. We do speculate that this is due to a completely different type of self-organization of the cardiac system of person #3. In other words, the dynamics of the cardiac system of person #3 experiences the collapse of complexity until the small jump before the 500-th contraction. This jump could be probably characterized as a partial AnT. Later (after the 1000-th contraction), person #3 experiences a complete AnT. It appears that the transition through the AnT is a very complex and individual process, which can vary from one person to another.

**Fig 4:**
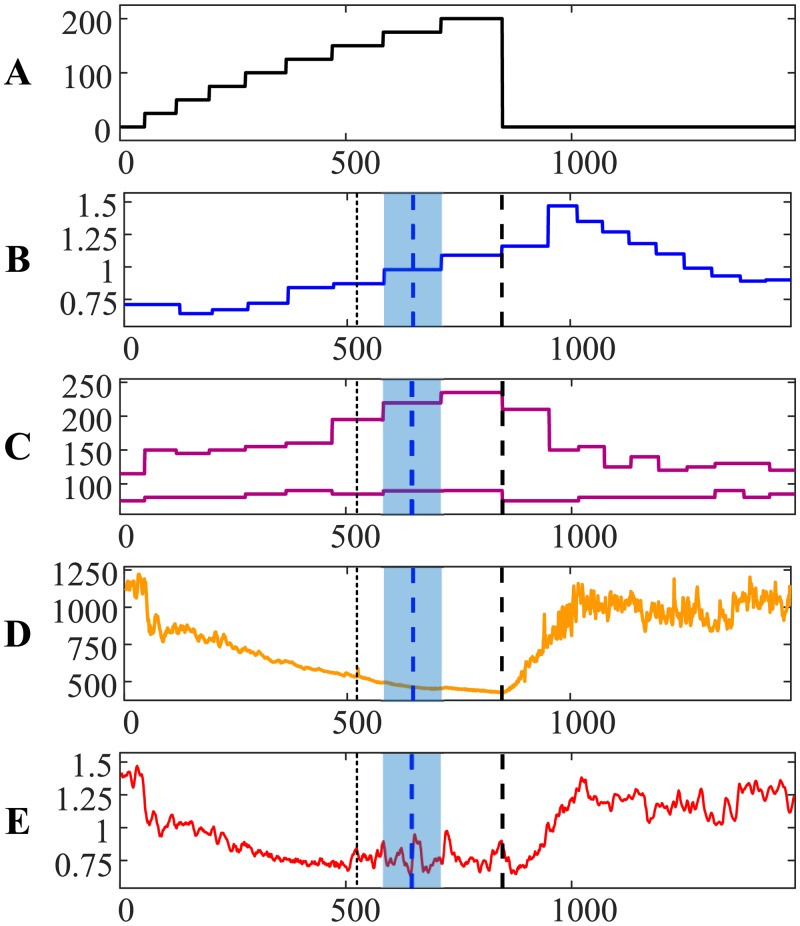
The transit through the AnT—Person #4.

Person #4 (Fig 4) is another example demonstrating an almost immediate drop of systolic and diastolic pressures immediately after the termination of the load. However, the AnT based on ECG is much less expressed for this person if compared to persons #1, #2 and #3. Note that higher numerical values of the algebraic relationship between two time series denote smaller similarity between these two time series (and vice versa) [21]. Post-anaerobic dynamics of person #4 is also characterized by strong fluctuations (Fig 4E)—but RR and QRS duration intervals are more interlinked if compared to other persons. The difference between the AnT based on ECG and the AnT based on exhaled air is minus 1.0058 min.

The ability to quantify the similarity between two distinct time series provides interesting insight to the dynamics of self-organization for person #3 during the recovery process (Fig 3E). Cardiac intervals RR and QRS duration are still strongly linked after the recovery. However, note that the heart rate of person #3 after the recovery did not return to the normal rate before the load (Fig 3D). That is a probable marker for some problems with the self-organization of the cardiac system. The situation is completely different with person #4—the heart rate returned to normal after the recovery (Fig 4D).

**Fig 5:**
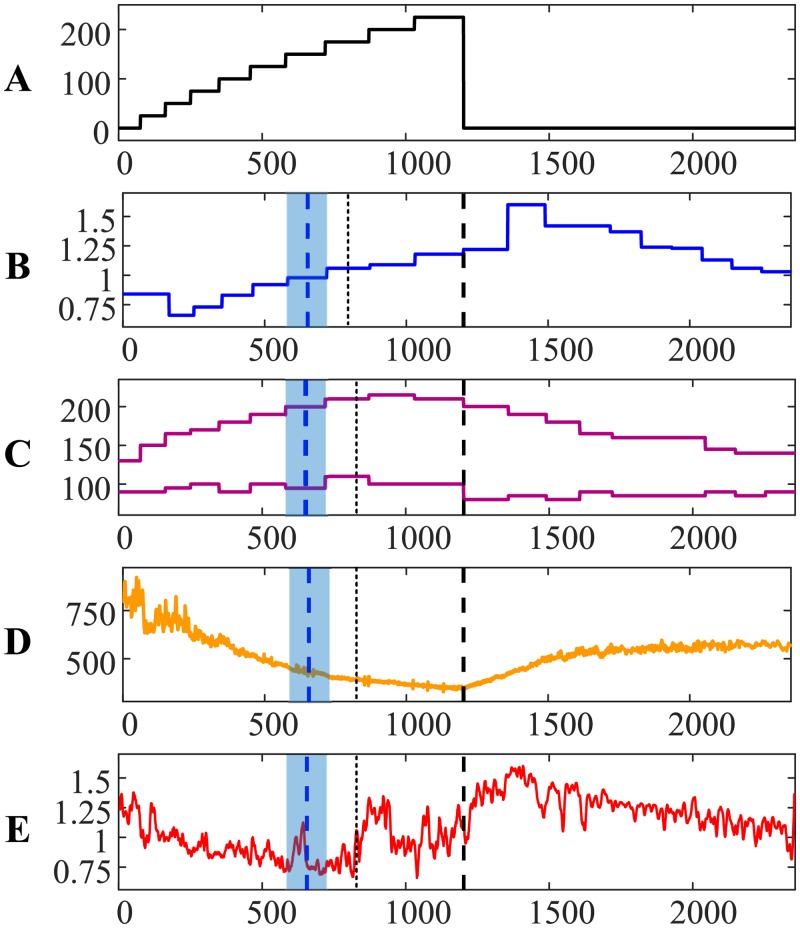
The transit through the AnT—Person #5.

The dynamics of self-organization of the person #5 is again different from all previous persons (Fig 5E). The decline of the algebraic relationship between RR and QRS duration intervals is non-monotonous. A sharp peak coincides with the AnT based on the exhaled air—but the relationship continues to decline until the AnT based on the ECG (Fig 5E). The difference between the AnT based on ECG and the AnT based on exhaled air is 1.2188 min. The recovery processes for person #5 and person #3 are similar.

**Fig 6:**
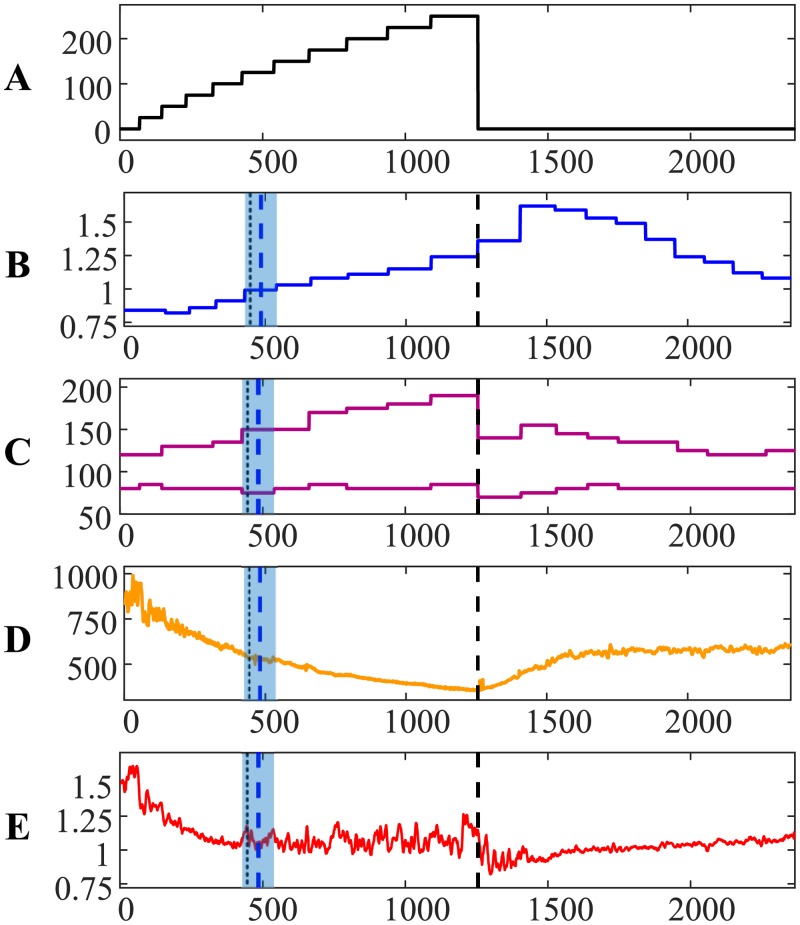
The transit through the AnT—Person #6.

The dynamics of person #6 is similar to person #4—except that the heart rate of person #6 does not return to normal after the recovery (Fig 6). The difference between the AnT based on ECG and the AnT based on exhaled air is minus 0.3514 min (Fig 6E).

**Fig 7:**
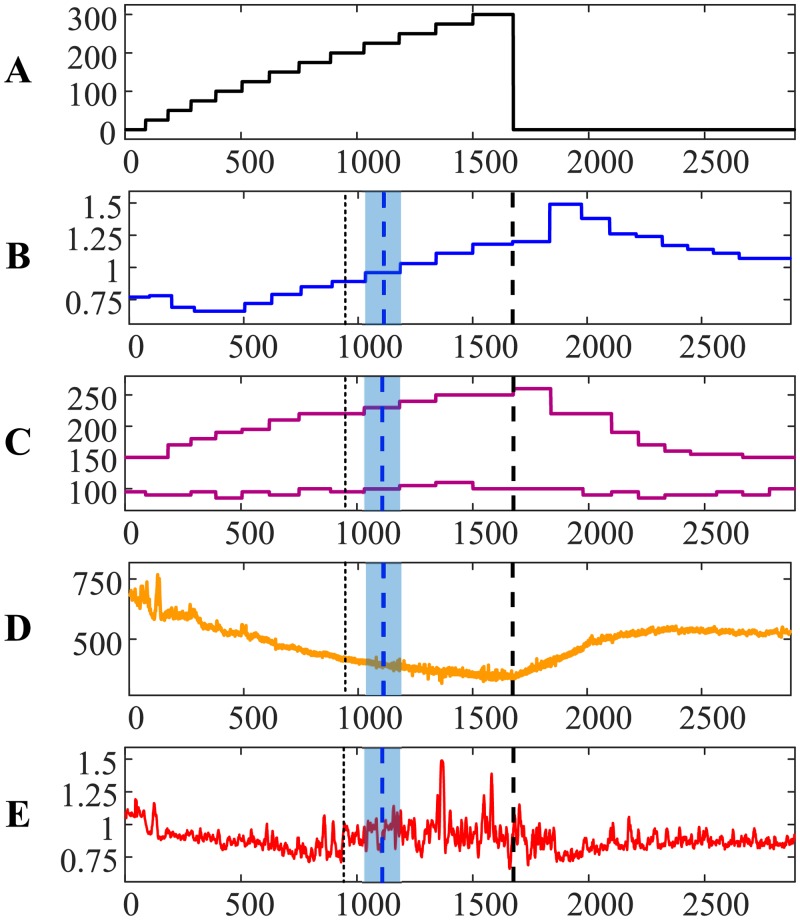
The transit through the AnT—Person #7.

The dynamics of person #7 is somewhat similar to person #6—however systolic and diastolic pressures do not drop immediately after the load for person #7 (Fig 7C) and the heart rate does not return to normal until the end of the recovery process (Fig 4D). The difference between the AnT based on ECG and the AnT based on exhaled air is minus 1.0917 min (Fig 7E).

**Fig 8:**
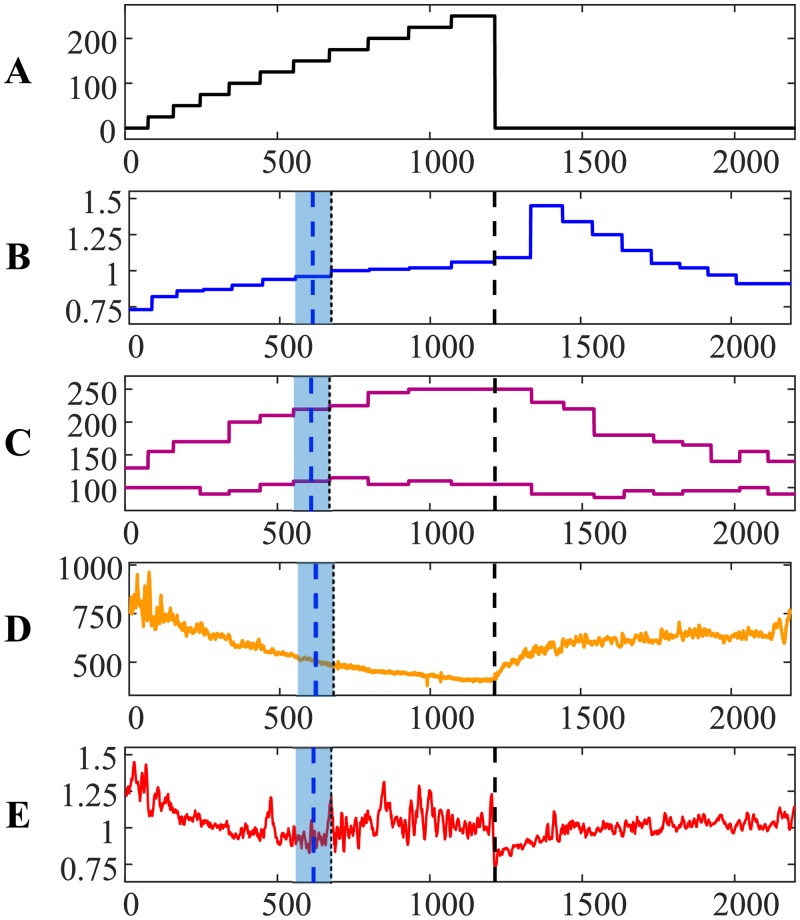
The transit through the AnT—Person #8.

The dynamics of person #8 is somewhat similar to person #5. Some intermittent peaks precede the AnT (Fig 8E). The difference between the AnT based on ECG and the AnT based on exhaled air is minus 0.5420 min (Fig 8E).

**Fig 9:**
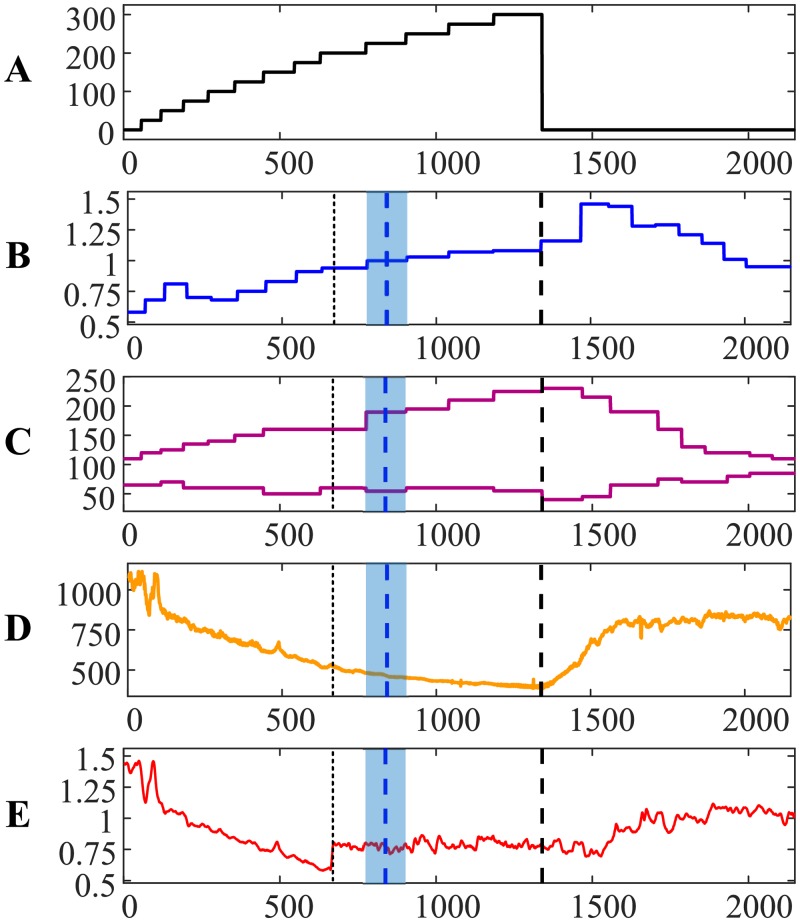
The transit through the AnT—Person #9.

The dynamics of person #9 is unique again. The AnT based on ECG is clearly indicated by a sudden jump (Fig 9E). However, the post-anaerobic variability of the relationship between RR and QRS duration intervals is much lower if compared to all other persons. The difference between the AnT based on ECG and the AnT based on the exhaled air is minus 1.3588 min (Fig 9E).

As mentioned previously, the CO_2_/O_2_ ratio in the exhaled air cannot be considered as an accurate indicator of the AnT. Therefore, the computed differences between the AnT based on ECG and the AnT based on the exhaled air can be used for the principal validation of the proposed technique—but not for the determination of its accuracy.

## Discussion

As mentioned previously, the main objective of this paper is to present a technique for the identification of the AnT based on the ECG analysis. It is well known that the AnT is a complex process—and varies according to individual physiological features of a person. However, the ability to track and observe the dynamics of the cardiac system allows not only to identify the AnT—but also to characterize transient processes of self-organization.

The first observation is that the algebraic relationship between RR and QRS duration intervals (Eq 1) can characterize the self-organization of the heart system before, during and after the load. This relationship can be used to illustrate the ability of the heart system to transit to different states (attractors). The presented visualization technique is in stark contrast with other available methods used to identify the AnT (based on the CO_2_/O_2_ ratio in the exhaled air, the concentration of lactate in the blood, the variation of the RR during the load).

The second observation is that the RR-QRS relationship reveals completely different features compared to the relationship between RR and JT intervals [21]. The RR-JT relationship does quantify the complexity in the self-organization of the heart system during the load (the collapse of complexity occurs at the end of the cycle ergometry exercise). On the contrary, the RR-QRS relationship reveals the self-organization of the heart system during the transit through the AnT.

The third observation is that the presented relationship can be used for the classification of the individual performance under the load. Moreover, the RR-QRS relationship could be used as a stand-alone indicator (without any other measurements except the ECG) for both the determination of the AnT and the characterization of the performance of the person during the load and the recovery. This is a major advantage compared to other existing techniques for the determination of the AnT. The presented technique is based on the measurement of standard cardiac intervals and does not require taking multiple blood samples during the exercise or complex experimental setups with masks and gas analyzers.

However, the RR-QRS relationship does not serve as a biomarker which could detect such conditions as atrial fibrillation, supraventricular tachycardia, or sudden cardiac death (by the way, this is also true for the RR-JT relationship in [21]). Instead, we propose a new visualization technique which can be beneficial for the characterization and the classification of the transit through the AnT. However, the development of pattern classification algorithms for automatic analysis of transient processes based on the RR-QRS relationship and the development of an automatic expert system for performance evaluation remains a definite objective of future research.

Finally, it would be interesting to understand what is the physiological mechanism that would explain that the sudden increase in the relationship between RR and QRS intervals is the AnT. However, as mentioned previously, the main objective of this paper is not to develop a new biomarker. This paper raises a hypothesis and provides a convincing evidence that the relationship between RR and QRS can be used as a new visualization technique for the characterization of the AnT. An important question about the relationships between other different cardiac intervals (and the interpretation of these relationships) remains open. That also remains a solid objective of future research.

## Conclusions

The presented algebraic relationship between RR-QRS cardiac intervals serves as an indicator for both the determination of the anaerobic threshold and the characterization of the performance of the person during the load and the recovery processes. All persons (#1—#9) were healthy. Another, and even more important objective would be the ability to perform early disease diagnosis for persons with different health disorders. That also remains one of the fundamental objectives of future research.

## Supporting information

S1 Dataset Data files. The supporting information files include data from ECG analysis for all persons.
(ZIP)
